# CysLT_1_R Antagonists Inhibit Tumor Growth in a Xenograft Model of Colon Cancer

**DOI:** 10.1371/journal.pone.0073466

**Published:** 2013-09-05

**Authors:** Sayeh Savari, Minghui Liu, Yuan Zhang, Wondossen Sime, Anita Sjölander

**Affiliations:** Division of Cell and Experimental Pathology, Department of Laboratory Medicine, Lund University, Skåne University Hospital, Malmö, Sweden; University of North Carolina at Chapel Hill, United States of America

## Abstract

The expression of the inflammatory G-protein coupled receptor CysLT_1_R has been shown to be upregulated in colon cancer patients and associated with poor prognosis. The present study investigated the correlation between CysLT_1_R and colon cancer development *in vivo* using CysLT_1_R antagonists (ZM198,615 or Montelukast) and the nude mouse xenograft model. Two drug administration regimens were established. The first regimen was established to investigate the importance of CysLT_1_R in tumor initiation. Nude mice were inoculated with 50 µM CysLT_1_R antagonist-pretreated HCT-116 colon cancer cells and received continued treatment (5 mg/kg/day, intraperitoneally). The second regimen aimed to address the role of CysLT_1_R in tumor progression. Nude mice were inoculated with non-pretreated HCT-116 cells and did not receive CysLT_1_R antagonist treatment until recordable tumor appearance. Both regimens resulted in significantly reduced tumor size, attributed to changes in proliferation and apoptosis as determined by reduced Ki-67 levels and increased levels of p21^WAF/Cip1^ (*P*<0.01), cleaved caspase 3, and the caspase-cleaved product of cytokeratin 18. Decreased levels of VEGF (*P*<0.01) and reduced vessel size (*P*<0.05) were also observed, the latter only in the ZM198,615-pretreatment group. Furthermore, we performed a series of *in vitro* studies using the colon cancer cell line HCT-116 and CysLT_1_R antagonists. In addition to significant reductions in cell proliferation, adhesion and colony formation, we observed induction of cell cycle arrest and apoptosis in a dose-dependent manner. The ability of Montelukast to inhibit growth of human colon cancer xenograft was further validated by using two additional colon cancer cell lines, SW-480 and HT-29. Our results demonstrate that CysLT_1_R antagonists inhibit growth of colon cancer xenografts primarily by reducing proliferation and inducing apoptosis of the tumor cells.

## Introduction

Eicosanoids include a wide variety of bioactive lipid metabolites derived from polyunsaturated 20-carbon essential fatty acids. Arachidonic acid belongs to the omega-6 family and is the precursor of eicosanoids such as prostanoids, leukotrienes, hydroxyl eicosatetraenoic acids (HETEs), and epoxides. These eicosanoids are considered pro-inflammatory; epidemiological, clinical, and laboratory studies have established that the aberrant metabolism of arachidonic acid via the cyclooxygenase (COX) and the lipooxygenase (LOX) pathways, which generate prostanoids and leukotrienes, respectively, can promote chronic inflammation and carcinogenesis [1], [2]. The unstable leukotriene A_4_ (LTA_4_) is formed by 5-LOX in the presence of 5-lipoxygenase-activating protein (FLAP). LTA_4_ is further metabolized to either LTB_4_ or the cysteinyl leukotrienes, LTC_4_, LTD_4_, and LTE_4_ [3].

Cysteinyl leukotrienes are involved in airway processes, such as mucus secretion, increased vascular permeability, eosinophil chemotaxis, and bronchoconstriction [4], [5], [6], [7]. Cysteinyl leukotrienes are also implicated in chronic inflammatory conditions, such as rheumatoid arthritis, asthma, and inflammatory bowel diseases (IBD) [8], [9], [10]. The inflammatory milieu has been widely appreciated as one of the enabling characteristics of cancer [11]. Accordingly, there is a strong correlation between long-standing IBD, such as ulcerative colitis and Crohn’s disease, in which pro-inflammatory eicosanoids (i.e., arachidonic acid derivates) are abundant and colorectal cancer [12], [13]. Colorectal cancer is the third most commonly diagnosed cancer in the world and has the fourth highest mortality rate [14]. It is estimated that patients suffering from IBD have an approximately 30-fold increased risk of developing colorectal cancer [15]. Other eicosanoids derived from the arachidonic pathway that are implicated in colon cancer include the prostanoids. Prostaglandin E_2_ (PGE_2_) is derived from arachidonic acid via the COX pathway and is the most abundant and most extensively studied prostanoid in cancer, especially colon cancer. PGE_2_ has been shown to increase tumor burden in the intestines of both APC ^Min/+^ and azoxymethane induced mice [2]. LOX-5 and COX-2, the enzymes responsible for producing cysteinyl leukotrienes and PGE_2_, respectively, have also been implicated in colon cancer. Their increased expression has been documented in patients with colorectal adenocarcinomas [16].

Cysteinyl leukotrienes mediate their effects through G-protein coupled receptors (GPCRs) and are referred to as CysLT_1_R and CysLT_2_R, based on their pharmacological characterization and functional profiling in response to a series of agonists or antagonists in different cellular and tissue systems [17]. CysLT_1_R has a higher affinity for LTD_4_, the most potent cysteinyl leukotriene, whereas CysLT_2_R has a lower but equal affinity for both LTD_4_ and LTC_4_ [18], [19]. ZM198,615 and Montelukast are selective CysLT_1_R antagonists used in studies of inflammatory diseases such as rheumatoid arthritis and asthma [20], [21]. The latter CysLT_1_R antagonist is also used in the clinic to treat asthmatic patients [22].

The balance between the CysLT_1_ and CysLT_2_ receptor seems to be important in the disease etiology of colon cancer. In fact, we have shown that these two receptors are co-localized and form both hetero-and homodimers in the human intestinal epithelial cell line Int 407 and that LTC_4_ stimulation of CysLT_2_R negatively regulates the cell surface expression of CysLT_1_R [23]. Our previous studies have also shown that LTD_4_, via CysLT_1_R induces the upregulation of proteins associated with colon cancer, such as COX-2, β-catenin, and Bcl-2 in intestinal epithelial cells [24]. In addition, we have shown that CysLT_1_R is upregulated in colon cancer patients and is associated with poor prognosis [16], whereas the concomitant low expression of CysLT_1_R and high expression of CysLT_2_R mediate good prognosis [25]. Moreover, our previous work has shown that LTD_4_-induced CysLT_1_R signaling results in cell proliferation, survival, and migration [26], [27]. In contrast, LTC_4_ stimulation of CysLT_2_R has been shown to induce the differentiation of colon cancer cells, and reduced expression of CysLT_2_R is associated with poor patient prognosis [28].

In the present study, we investigated the function of CysLT_1_R in colon cancer growth using CysLT_1_R antagonists. The effects of CysLT_1_R antagonists on HCT-116 human colon cancer cells were studied both *in vitro* and *in vivo* using the nude mouse xenograft model.

## Materials and Methods

### Reagents

The CysLT_1_R antagonist ZM198,615 (ICI-198,615) was a gift from AstraZeneca and the CysLT_1_R antagonist Montelukast was purchased from Cayman Chemicals Co. (Ann Arbor, MI). Cell proliferation reagent WST-1 and the mouse monoclonal M30 CytoDEATH antibody (1∶10) were from Roche (Basel, Switzerland). The Annexin V-PE Apoptosis Detection Kit was from BD Pharmingen (San Diego, CA). The Quick Start™ Bradford Dye Reagent, Mini-PROTEAN TGX™ Gels, secondary horseradish-conjugated antibodies, and chemiluminescent detection reagent were from Bio-Rad Laboratories (Hercules, CA). Rabbit monoclonal anti-human cleaved caspase 3 antibody (1∶200) was purchased from Cell Signaling Technology (Danvers, MA). The rabbit monoclonal anti-human Ki67 antibody (1∶500) was obtained from Thermo Fisher Scientific (Waltham, MA). Goat polyclonal anti-mouse PECAM-1 (CD31) antibody (1∶700) and rabbit polyclonal anti-human VEGF antibody (1∶200) were purchased from Santa Cruz Biotechnology (Santa Cruz, CA). Mouse monoclonal anti-human p21^WAF1/Cip1^ antibody (1∶1200) was from DakoCytomation (Glostrup, Denmark). Rabbit polyclonal anti-human CysLT_1_R antibody (1∶250) was obtained from Innovagen (Lund, Sweden). Mouse monoclonal anti-β-actin antibody was from Sigma Chemical Co. (St. Louis, MO). The Cysteinyl Leukotriene EIA kit was purchased from Cayman Chemical Company (Ann Arbor, MI). All other chemicals were of analytical grade and were obtained from Chemicon International (Temecula, CA) or Sigma Chemical Co. (St. Louis, MO).

### Cell Culture

HCT-116 cells (ATCC^®^ No. CCL-247), derived from human colon carcinoma, SW-480 (ATCC^®^ No. CCL-228) and HT-29 (ATCC^®^ No. HTB-38), derived from human colon adenocarcinoma, were obtained from the American Type Culture Collection (Manassas, VA).

HCT-116 and HT-29 cells were cultured in McCoy’s 5A medium, while SW-480 cells were cultured in RPMI 1640. All media was supplemented with 10% fetal bovine serum (FBS) 55 µg/ml streptomycin, 55 IU/ml penicillin, and 1.5 µg/ml fungizone. The cells were grown for 5 days to 70*–*80*%* confluence at 37°C in a humidified atmosphere of 5% CO_2_. All experiments were conducted with cells at passages 5 to 30, and the cells were regularly tested to ensure the absence of mycoplasma contamination.

### Tumor Xenograft Studies

**Figure 1:**
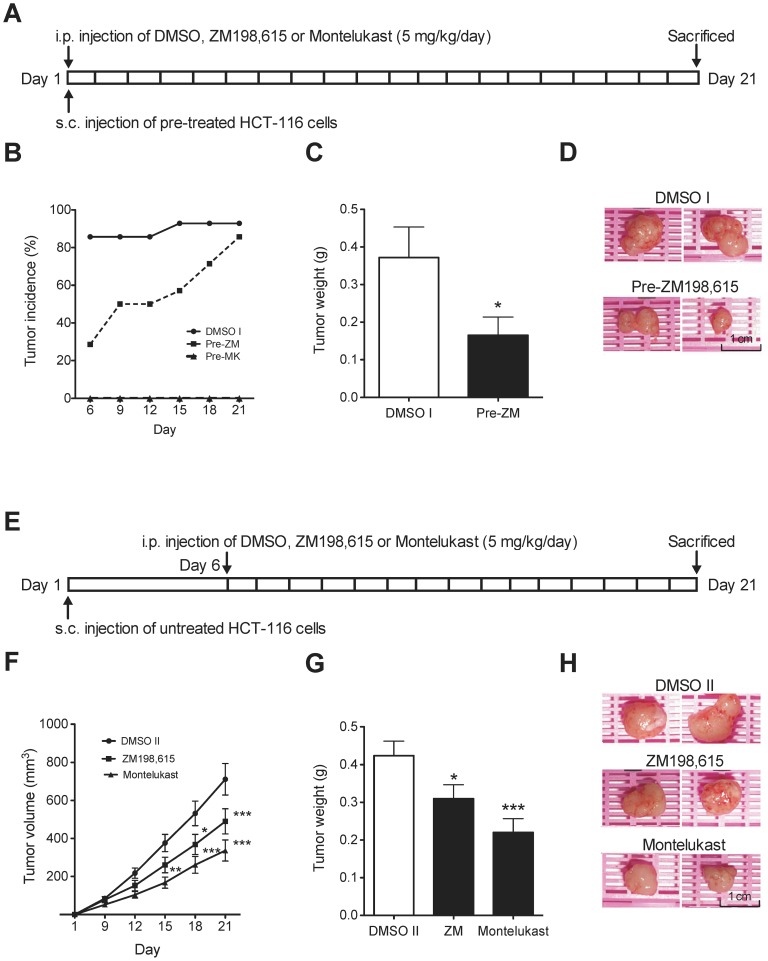
Effects of CysLT_1_R antagonists on HCT-116 xenograft tumor growth. (**A**) Experimental protocol for the pretreatment groups; BalbC (nu/nu) mice were subcutaneously injected into two flanks with HCT-116 cells pretreated with ZM198,615 or Montelukast (50 µM), and received treatment intraperitoneally from the day of inoculation with DMSO, ZM 198.615, or Montelukast (5 mg/kg/day). (**B**) Tumor incidence of mice treated with DMSO (DMSO I group), ZM198,615 (Pre-ZM group), or Montelukast (Pre-Montelukast group) and (**C**) tumor weight compared to the DMSO I group at the end of the experiment (day 21). (**D**) Representative tumor images from the pretreatment group. (**E**) Experimental protocol for the treatment study; non-pretreated HCT-116 cells were subcutaneously injected into two flanks of nude mice. DMSO (DMSO II group), ZM198,615 (ZM group), or Montelukast (Montelukast group) treatment began on day 6 after tumor cell inoculation. (**F**) Tumor volumes over a 21-day period and (**G**) tumor weight at the end of the experiment (day 21). (**H**) Representative tumor images from the treatment group. The quantitative data shown are the mean ± SEM. *P<0.05, **P<0.01, ***P<0.001. Tumor volume analysis was performed by two-way ANOVA and tumor weight analysis was performed by Student’s *t* test.

All animal experiments were approved by the Regional Ethical Committee for Animal Research at Lund University, Sweden (M205-10). Female 6-to 8-week-old athymic nude mice (BalbC nu/nu) were purchased from Taconic Europe A/S (Ry, Denmark). To induce subcutaneous human colon cancer xenografts, 2.5 ×10^6^ low-passage HCT-116 cells in 100 µl PBS were injected into two flanks per mouse (n = 7 mice/group). Two drug administration regimens were established to investigate the functional importance of CysLT_1_R antagonists in tumor initiation and progression. Animals treated according to the first regimen were inoculated with HCT-116 cells pretreated with either DMSO (DMSO I), 50 µM ZM198,615 (Pre-ZM), or 50 µM Montelukast (Pre-Montelukast), for 30 min. Cell viability was determined by trypan blue dye exclusion assay and only viable cells were considered for subcutaneous injection. Thereafter, from the day of inoculation, mice received daily i.p. injections with DMSO or CysLT_1_R antagonists (5 mg/kg) dissolved in DMSO, diluted in PBS for a total volume of 100 µl (Figure 1A). According to the second regimen, animals were inoculated with non-pretreated HCT-116 cells. Once palpable tumors were established 6 days after injection, the mice were randomly divided into three groups and then decided which group should be treated with DMSO (DMSO II), ZM198,615, or Montelukast. The mice received daily i.p. injections of DMSO or the CysLT_1_R antagonists (5 mg/kg) (Figure 1E).

In addition, SW-480 or HT-29 cells, 2.5×10^6^ low-passage cells in 100 µl PBS were injected into two flanks per mouse (n = 12 and n = 18 mice for SW-480 and HT-29, respectively) to induce subcutaneous human colon cancer xenografts in female 6-to 8-week-old athymic nude mice (BalbC nu/nu). All mice had established palpable tumors in both flanks at day 7 and were randomized into two groups for each cell line. Before initiating the treatments, one investigator measured the tumor sizes of all tumors to secure that there was no size difference in between the different groups. The mice then recieved daily i.p injections for 14 days with either DMSO or Montelukast (5 mg/kg) ( = 6 and n = 9 mice per treatment group for SW-480 and HT-29, respectively).

Mouse body weight and tumor size were recorded every third day. The formula for calculating tumor volume was *V* = π/6 ×1.58 (*length × width*)^3/2^ [29]. After 21 days, all mice were sacrificed, and the tumors removed, measured, weighed, and photographed. Tumor tissues were fixed in 10% buffered formalin, embedded in paraffin for immunohistochemistry analysis and/or snap frozen in liquid nitrogen, and stored at −80°C for Western blot analysis.

The dose of Montelukast (5 mg/kg) was chosen on the basis of published data, where dosages ranging from 5–10 mg/kg have been reported in a wide range of mice experimental models [30], [31], [32]. The dosages of 2 mg/kg and 10 mg/kg were also investigated and the results reveal similar tendencies in xenograft tumor growth inhibition (Data not shown).

### Immunohistochemistry

Paraffin-embedded sections obtained from xenografted tumors were sectioned (5 µm) for immunohistochemical staining. All procedures were performed using a Dako automatic slide stainer according to the manufacturer’s instructions. The slides were photographed with a Nikon Eclipse 800 microscope and evaluated in a blinded fashion by two observers independently. Entire Ki-67 stained sections were scanned with Aperio ScanScope CS (Aperio Technologies, Inc, Vista, CA) and an area in which staining was particularly prevalent (i.e., hot spot) was identified in each tumor using a low-power field (×40). 3 high-power field (×400) images were selected for analysis in each hot spot. Using NIS-Elements software a threshold was set to define and measure ratio of Ki-67 positive stained area to the total high-power field area. Estimation of apoptotic cells was performed by detection of the caspase-cleaved product of cytokeratin 18 with CytoDEATH (M30). Depending on tumor size, 5–10 random fields were chosen, and the average apoptotic cell number per field was measured (×200). Antibody directed against CD31 was used to quantify microvessel density (MVD). Images (×100) were taken from three areas with the highest microvessel density appearance (i.e., hot spots) and the mean value of CD31-positive counts calculated. To estimate the area of CD31-positive structures (vessel area), the images were saved as TIFF files. Positive staining was quantified using the Adobe Photoshop threshold function and combined with histogram analyses. The mean number of positive pixels per tumor section from three hot spots was recorded.

### Western Blot

For cleaved caspase 3 and CysLT_1_R analyses, the cells were cultured for 5 days to 70% confluence. Only adherent HCT-116, SW-480 and HT-29 cells were collected for CysLT_1_R analyses. For the analysis of cleaved caspase 3 we used both adherent and floating HCT-116 cells. Cell lysates were prepared and solubilized in sample buffer as previously described [26]. Protein extraction from xenografted tumor tissue was performed by sonication. Briefly, tumor tissues in 700 µl ice-cold reducing loading buffer (62.5 mM Tris, pH 6.8, 6 M urea, 10% glycerol, and 2% SDS) containing protease inhibitors (2 mM Na_3_VO_4_, 4 µg/ml leupeptin, and 60 µg/ml phenylmethylsulfonyl fluoride) were subjected to sonication on ice for 30 sec. Whole-cell lysates were centrifuged at 3400×*g* for 15 min at 4°C. Bromophenol blue (0.003%) and mercaptoethanol (5%) were added to the sample supernatants. Proteins were separated by electrophoresis on precast any kD™ SDS-polyacrylamide gels and electrotransferred onto PVDF membranes. Membranes were blocked with either 5% nonfat dry milk or 5% BSA in 0.05% Tween/PBS for 1 h at room temperature and then incubated with primary antibody overnight at 4°C. Finally, the membranes were incubated with an appropriate secondary antibody conjugated with horseradish peroxidase for 1 h at room temperature and detected with a chemiluminescence reagent. Immunoblotting results were visualized with the Molecular Imager ChemiDoc XRS System and Image Lab software (Bio-Rad Laboratories).

### Proliferation Assay

Cell proliferation was measured by using the WST-1 cell proliferation assay according to the manufacturer’s instructions. Briefly, cells were seeded in triplicate in flat-bottomed 96-well plates at 1,500 cells/well and grown for 24 h in medium containing 2% FBS. Thereafter, cells were treated with CysLT_1_R antagonists for different time points. After incubation with 10 µl of WST-1 reagent for 90 min, the absorption of the samples was measured at 440 nm using the Tecan Infinite M200 plate reader.

### Flow Cytometry

Cell cycle and cell death measurements were assessed with flow cytometry. Briefly, HCT-116 cells were serum-starved overnight and treated with CysLT_1_R antagonists in fresh medium containing 2% FBS. After 24 h, adherent and floating cells were harvested and washed with PBS. For cell cycle profiles, cells were immediately fixed in 70% (v/v) ethanol, treated with 0.1% sodium citrate and 100 µg/ml RNase A, and incubated for 30 min at 37°C with 50 µg/ml propidium iodide. Induction of apoptosis was determined in viable cells using the Annexin V-PE Apoptosis Detection Kit according to manufacturer’s protocol. All flow cytometric measurements were performed using the FACS Calibur flow cytometer (Becton Dickinson, San Jose, CA), and analyses were performed using FCS Express, version 4.0 (De Novo Software).

### Adhesion Assay

HCT-116 cells were suspended in medium containing 2% FBS at a density of 2.0×10^5^ cells/ ml and treated with or without CysLT_1_R antagonists for 30 min at 37°C before plating in flat-bottomed 12-well plates (Corning, 1 ml/well). Cells were incubated at 37°C in 5% CO_2_ for 1 h, followed by three washes with PBS to remove unattached cells. After fixation in 4% formaldehyde for 15 min, cells were washed twice with PBS and stained with crystal violet (5 mg/ml in 2% ethanol) for 10 min at room temperature. Next, cells were washed extensively, and staining was released using 2% SDS in PBS. The staining intensity was quantified by spectrophotometry at 550 nm using the Tecan Infinite M200 plate reader.

### Soft Agar Assay

HCT-116 cells were cultured in medium containing 2% FBS with or without CysLT_1_R antagonists. Briefly, 1 ml of 0.5% agar/well (bottom layer) was added to 6-well plates and allowed to solidify for at least 1 h at room temperature. Then, 1.0×10^4^ cells were suspended in 1 ml medium with 0.35% agarose (top layer). Different doses of CysLT_1_R antagonists were added to the agarose (the top layer) and agar (the bottom layer) before they were placed onto the wells. An additional 2 ml of culture medium containing the CysLT_1_R antagonists were placed above the top layer. The medium was replaced every 3 days with or without the addition of CysLT_1_R antagonists. After 14 days of incubation at 37°C, colonies were visualized by staining with 0.005% crystal violet. Images were acquired using the ChemiDoc™ XRS+ System and the colonies were counted using ImageJ software.

### Cysteinyl Leukotriene Enzyme Immunoassay

Cells were cultured for 5 days to 70–80% confluence. At day 4 the media was changed and collected at day 5 for cysteinyl leukotriene separation by solid-phase extraction Sep-Pak Vac RC (C18–500 mg) cartridges from Water Corporation (Milford, MA). Cysteinyl leukotriene production was measured with an enzyme immunoassay according to manufacturer’s instructions.

### Statistical Analysis

All statistical analyses were performed in Prism Software (GraphPad, Inc.), and the statistical significance of data was determined as *P*<0.05. For comparison between two groups, either a paired or unpaired *t* test (Student’s *t* test) was used. One-way or two-way ANOVA was used to compare multiple groups. All values are expressed as the mean ± standard error of the mean (SEM).

## Results

### CysLT_1_R Antagonists Decrease Xenograft Tumor Growth

A colon cancer xenograft model was employed to investigate the effects of CysLT_1_R antagonists on cancer growth *in vivo*. To examine the effects of CysLT_1_R antagonists on tumor initiation, we inoculated nude mice with HCT-116 cells pretreated with CysLT_1_R antagonists. Treatment was begun immediately with either ZM198,615 or Montelukast (5 mg/kg/day) on the day of inoculation. The mice were sacrificed on day 21, before tumor volumes reached 1 cm^3^, according to ethical permission (Figure 1A). As shown in Figure 1B, tumor occurrence was significantly delayed in the Pre-ZM group (4 tumors) compared to the DMSO I group (12 tumors) on day 6. Furthermore, Montelukast pretreatment completely inhibited HCT-116 tumor generation. The mean tumor weight was significantly reduced in the Pre-ZM group compared to the DMSO I group (0.165±0.048 g *vs.* 0.372±0.082 g; Figure 1C).

In addition, we examined the effects of CysLT_1_R antagonists on tumor progression by inoculating nude mice with non-pretreated HCT-116 cells. After recordable tumor initiation (on day 6), CysLT_1_R antagonist treatments were carried out for 2 weeks (Figure 1E). On day 21, the average tumor size of the ZM198,615 and Montelukast groups was significantly smaller than tumors in the DMSO II group (490.1±66.21 mm^3^ and 336.9±55.38 mm^3^ *vs.* 711.6±82.6 mm^3^, *P*<0.05 or *P*<0.001, respectively) (Figure 1F). Similarly, the average tumor weight in the ZM198,615 and Montelukast groups versus the DMSO II group was significantly reduced (Figure 1G; 0.31±0.037 g and 0.22±0.036 g *vs.* 0.424±0.038 g, respectively, *P*<0.05). Figure 1D and H are representative tumor images taken from each group. In conclusion, these results support the hypothesis that CysLT_1_R is important for colon cancer growth.

### CysLT_1_R Antagonists Reduce Proliferation and Induce Apoptosis

**Figure 2:**
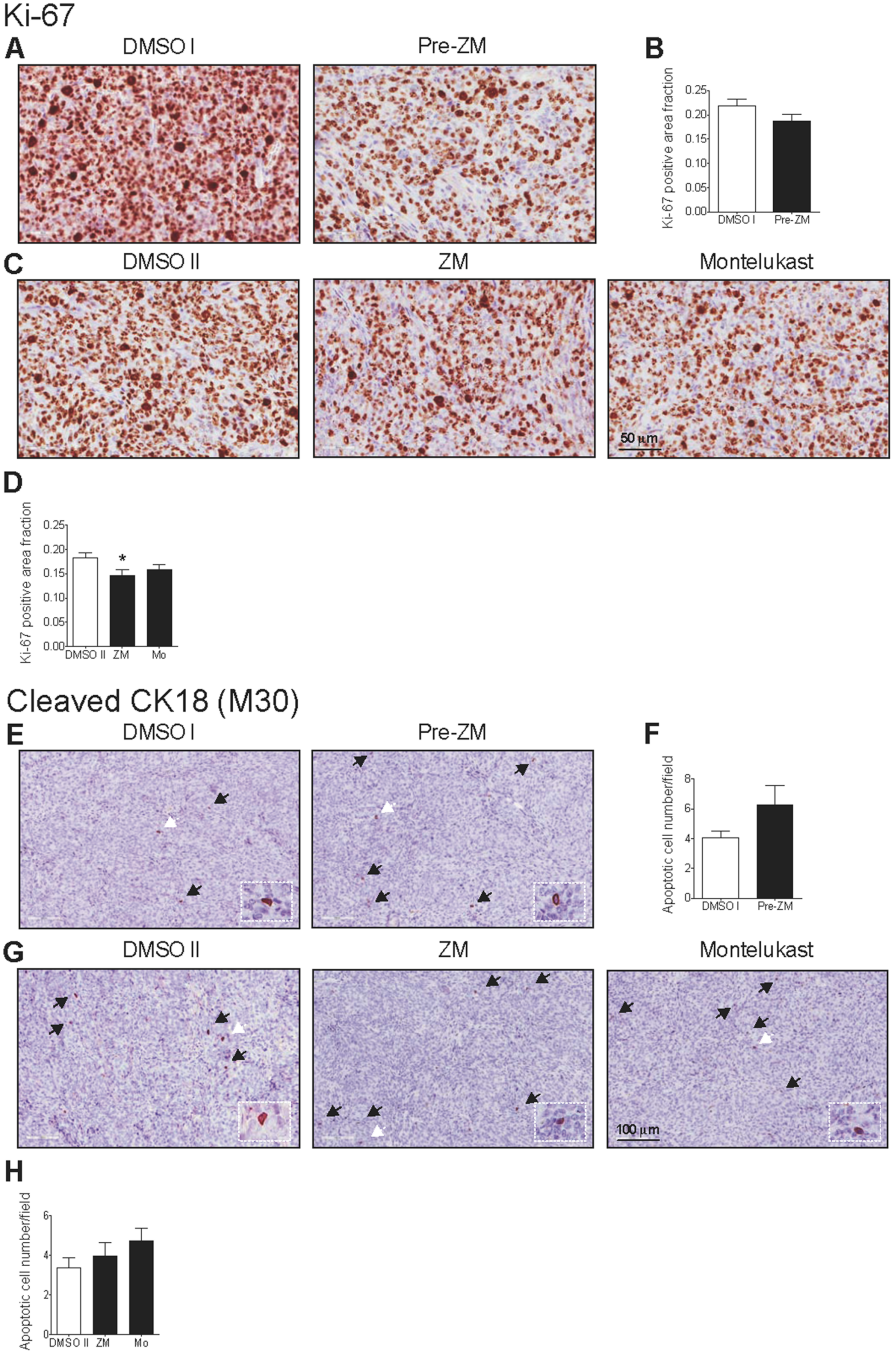
Effects of CysLT_1_R antagonists on HCT-116 xenograft tumor proliferation and apoptosis. (**A** and **C**) Representative Ki-67-stained images from paraffin sections of xenograft tumors (×400). (**B** and **D**) One Ki-67-stained hot spot was selected from each tumor and 3 separate areas within these hot spots were analyzed at high power field (×400). Ki-67 positive area fraction was determined as ratio of stained area to total high power field area. (**E** and **G**) Representative M30 CytoDEATH-stained images from paraffin sections of xenograft tumors (×200). Black and white arrows indicate positively stained cells. Boxed regions within the main panels shows the positively stained cells indicated by the white arrows at higher magnification (×400). (**F** and **H**) Average apoptotic cell number per field was determined by M30- positive counts (black arrows) in median-sized xenograft tumor sections taken from the middle part. The quantitative data shown are the mean ± SEM. *P<0.05 by Student’s *t* test.

We next investigated the underlying mechanisms by which CysLT_1_R antagonists exerted their inhibitory effects on tumor growth. HCT-116 tumor sections were stained with the proliferation marker Ki-67 or the apoptosis marker M30 CytoDEATH. The most prevalent Ki-67 stained area was selected for each xenograft tumor and three high power field images within this area were further analyzed. The Ki-67 level in these selected areas was moderately decreased in Pre-ZM group (Pre-ZM *vs.* DMSO I group; Figure 2A and B) and statistically significantly (*P<*0.05) decreased in treatment groups (ZM198,615 *vs.* DMSO II group; Figure 2C and D). Apoptotic cell number slightly increased in tumors from the Pre-ZM group (Pre-ZM *vs.* DMSO I group; Figure 2E and F) and the treatment groups (ZM198,615 or Montelukast *vs.* DMSO II group; Figure 2G and H).

**Figure 3:**
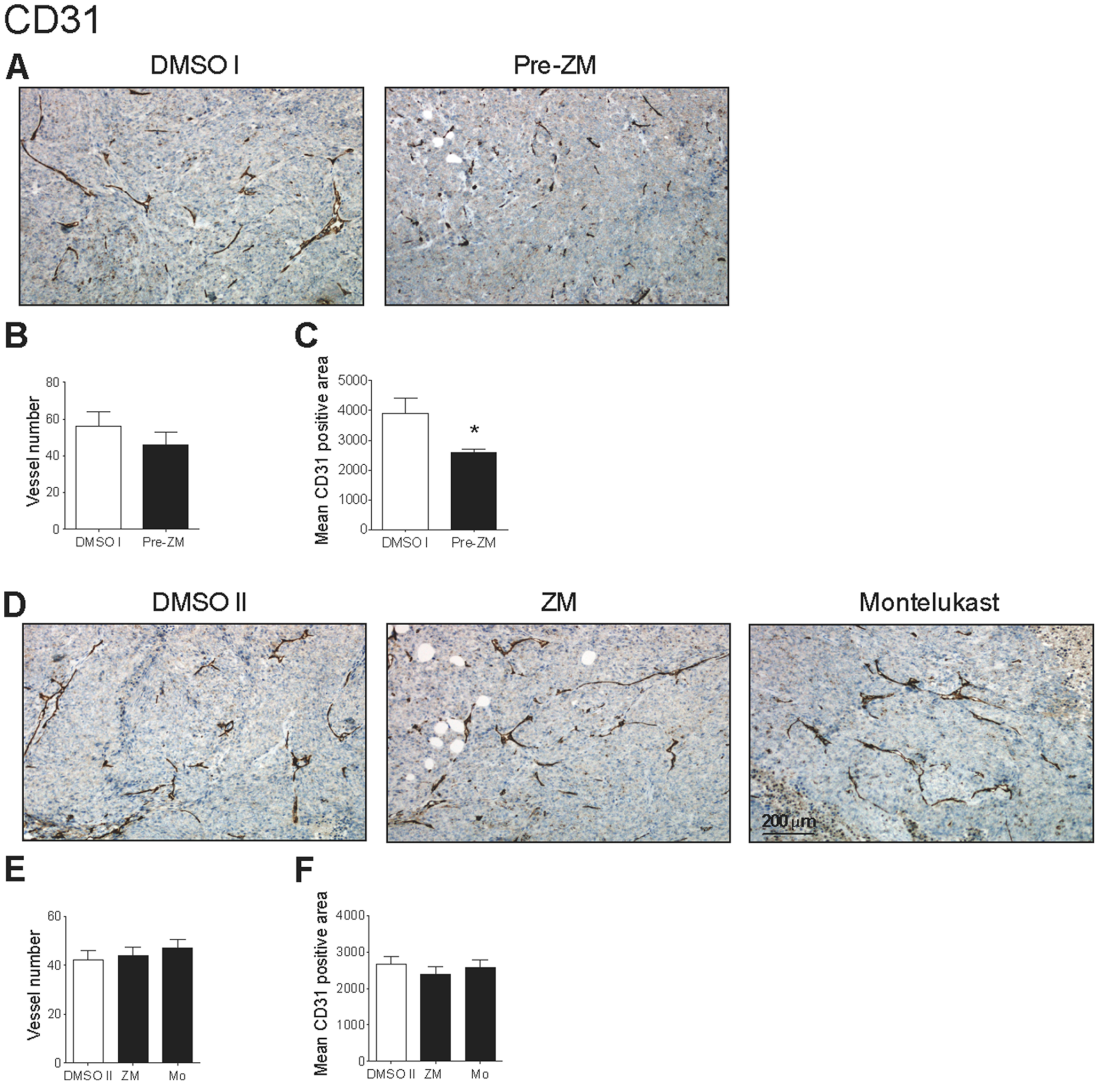
Effects of CysLT_1_R antagonists on HCT-116 xenograft tumor angiogenesis. (**A** and **D**) Representative CD31 stained images (×100). (**B** and **E**) Vessel density was determined with CD31-positive counts in three different fields (hot spots). (**C** and **F**) Quantitative analysis of CD31-positive areas using Adobe Photoshop. The quantitative data shown are the mean ± SEM. *P<0.05 by Student’s *t* test.

The effects of the CysLT_1_R antagonists on tumor vascularization were studied by staining for CD31, an endothelial cell-specific antigen. We observed a slightly decreased vessel number in the sections taken from the Pre-ZM group compared to the DMSO I group (46.1±6.7 *vs.* 56.0±7.9; Figure 3A and B). Vascular number is not the only parameter to indicate adequate tumor blood supply; vessel area is also a critical determinant of tumor blood flow [33]. In tumor sections from the Pre-ZM group, we noticed that the vessels appeared smaller and thinner, and had less branching. The tumor vessels in the DMSO I group appeared more mature with lumens, thick walls, and strong CD31 staining along their lengths. We therefore measured the CD31-positive staining areas. As shown in Figure 3C, tumors from the Pre-ZM198,615 group had a statistically significant (*P*<0.05) decreased mean of the CD31-positive area compared to tumors in the DMSO I group (2596±121.4 pixels *vs.* 3900±522.3 pixels, respectively), corresponding to a 33% reduction. There were no statistically significant differences in the mean number of vessels and vascular size among mice in the treatment groups (DMSO II *vs.* ZM198,615 or Montelukast; Figure 3D, E, and F). The reduced vascular size in the tumor sections taken from the Pre-ZM group indicated that CysLT_1_R antagonist treatment for 21 days could inhibit tumor vascularization and have a more pronounced effect on tumor progression.

**Figure 4:**
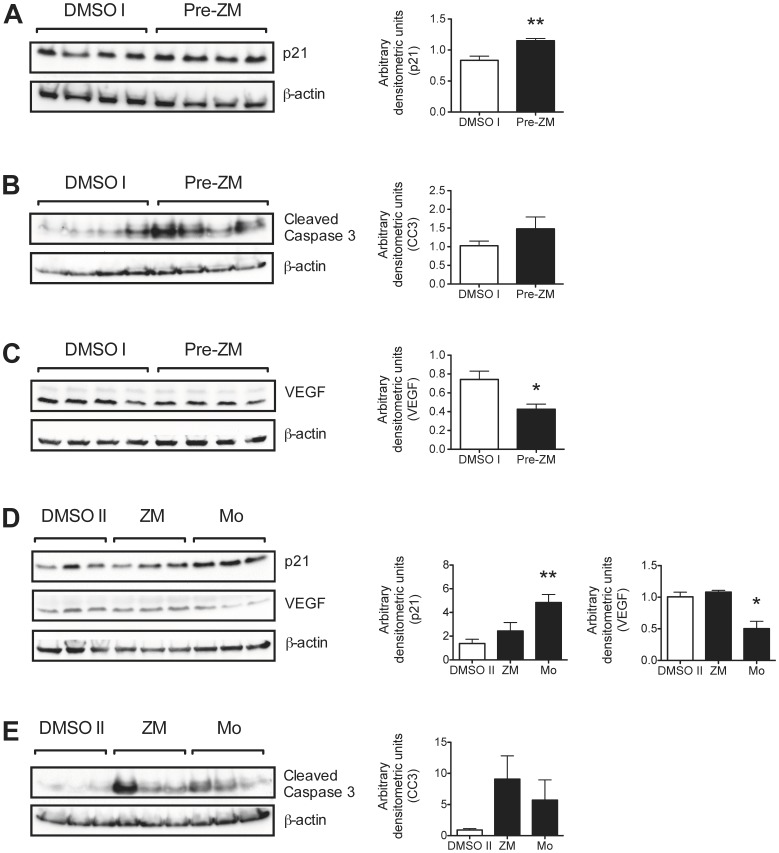
Effects of CysLT_1_R antagonists on cell cycle, apoptosis, and angiogenesis in HCT-116 xenograft tumors. Tumor samples (three or four tumors from each group) were subjected to Western blot analysis. Membranes were probed for (**A** and **D**) p21^ WAF/Cip1^; (**B** and **E**) cleaved caspase 3; and (**C** and **D**) VEGF. Data were normalized on the basis of β-actin levels**.** Densitometric analysis of protein expression represents the mean ± SEM. *P<0.05, **P<0.01 by Student’s *t* test.

Next, the expression levels of selected proteins involved in the cell cycle, apoptosis, and angiogenesis were investigated. p21^WAF/Cip1^, a potential cell cycle inhibitor, was shown to be significantly upregulated in tumor samples from the Pre-ZM group compared to the DMSO I group (*P*<0.01; Figure 4A). We also observed moderately increased levels of cleaved caspase 3 fragments (Figure 4B) and significantly decreased expression levels of VEGF (*P*<0.05; Figure 4C) in tumors from the Pre-ZM group compared to the DMSO I group. Similar analysis were made for the treatment groups (ZM198,615 or Montelukast *vs.* DMSO II). Significantly increased expression levels of p21^WAF/Cip1^ (*P*<0.01; Figure 3D) and decreased expression levels of VEGF (*P*<0.05; Figure 4D) could be observed for the Montelukast-treated group, but not for the ZM198,615-treated group compared to the DMSO II group. Increased levels of cleaved caspase 3 fragments were also observed in the treatment groups (ZM198,615 or Montelukast *vs.* DMSO II) (Figure 4E).

### CysLT_1_R Antagonists Reduce Proliferation and Induce G_1_ Arrest of HCT-116 Cells

**Figure 5:**
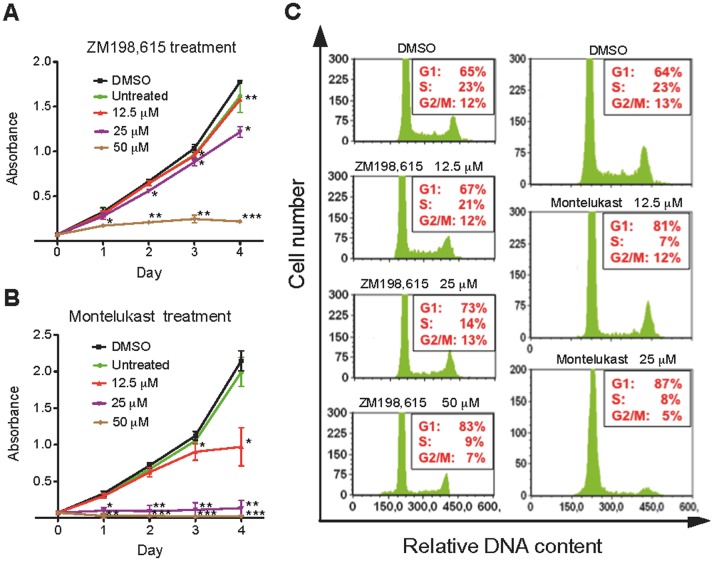
Effects of CysLT_1_R antagonists on HCT-116 cell proliferation and cell cycle. Cell proliferation was measured using the WST-1 cell proliferation assay, and absorption of the samples was measured at 440 nm. Cells were treated with (**A**) ZM198,615 or (**B**) Montelukast for 24, 48, 72, or 96 h. (**C**) Cell cycle analysis was carried out with propidium iodide staining. Percentages of the total cell population in different phases of the cell cycle were analyzed by flow cytometry. The quantitative data shown are the mean ± SEM from three separate experiments. *P<0.05, **P<0.01, ***P<0.001 by paired *t* test.

Because we observed that CysLT_1_R antagonist treatment could inhibit tumor growth *in vivo* partly by inducing cell cycle arrest, we were next interested to confirm this finding *in vitro*. To investigate whether CysLT_1_R antagonists had any direct effect on colon cancer cell proliferation, the WST-1 cell proliferation assay was performed. Treatment with increasing concentrations of ZM198,615 caused inhibition of HCT-116 cell proliferation in a dose-dependent manner. On day 4, the growth of cells treated with 12.5, 25 and 50 µM ZM198,615 was reduced by 11%, 31%, and 88% respectively, compared to DMSO-treated control cells (Figure 5A). When the same concentrations of Montelukast as ZM198,615 were used, we observed an even stronger effect on cell growth inhibition; 35%, 88%, and 100% for 12.5, 25, and 50 µM Montelukast, respectively, compared to the DMSO-treated control cells (Figure 5B). We next examined whether the CysLT_1_R antagonist-mediated growth inhibition of HCT-116 cells was due to cell cycle intervention. We found that Montelukast induced cell cycle arrest of HCT-116 cells within 24 h at G_1_ phase. As shown in Figure 5C, right panel, 81% and 87% of cells treated with 12.5 and 25 µM Montelukast, respectively, were in G_1_ phase compared to 64% of cells treated with DMSO. Similar results were observed for ZM198,615-treated cells (Figure 5C, left panel).

### CysLT_1_R Antagonists Induce Apoptosis of HCT-116 Cells

**Figure 6:**
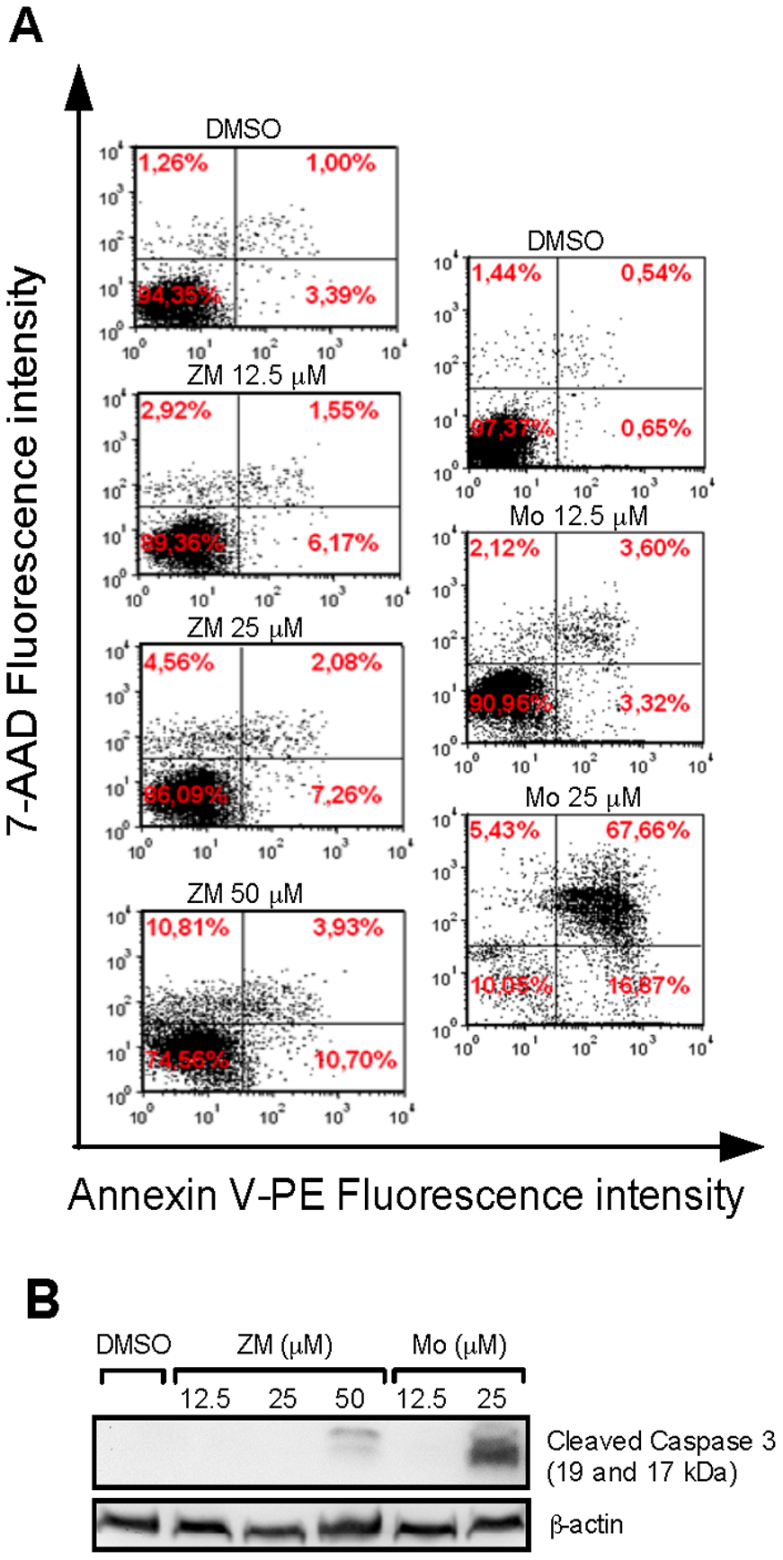
Effects of CysLT_1_R antagonists on apoptosis in HCT-116 cells. (**A**) Representative flow cytometry panels display apoptosis of HCT-116 cells treated with ZM198,615 (ZM) or Montelukast (Mo) using Annexin V-PE and 7-AAD-staining. (**B**) The level of cleaved caspase 3 fragments (19 and 17 kDa) in HCT-116 cells treated with CysLT_1_R antagonists as determined by Western blot analysis. Data shown are representative of three separate experiments.

Earlier studies have shown that Montelukast induces apoptosis in different prostate cancer cells [34]. Here, we evaluated whether CysLT_1_R antagonists could induce death of colon cancer cells. FACS analysis showed that both ZM198,615 and Montelukast could induce dose-related early and late apoptosis in HCT-116 cells (Figure 6A), and these data were supported by Western blot analyses (Figure 6B). ZM198,615- and Montelukast-treated cell lysates demonstrated a significant increase in cleaved caspase 3 fragments. These data suggest that CysLT_1_R antagonists induce apoptosis of HCT-116 cells and in accordance with our findings *in vivo*.

### CysLT_1_R Antagonists Reduce HCT-116 Cell Adhesion and Colony Formation

**Figure 7:**
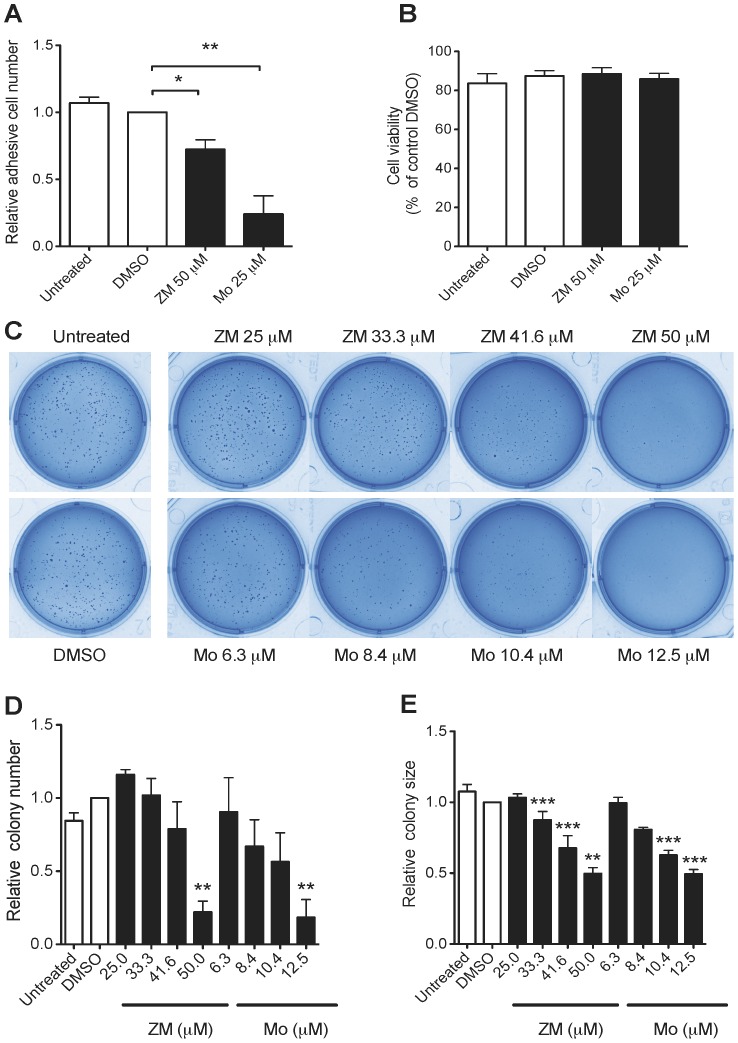
Effects of CysLT_1_R antagonists on HCT-116 cell adhesion and colony formation. (**A**) Briefly, HCT-116 cells were pretreated with ZM198,615 (ZM) or Montelukast (Mo) for 30 min, stained with 0.5% crystal violet and quantified with spectrophotometry at 550 nm. Relative adhesive cell number compared to the DMSO-treated control cells. (**B**) Cell viability as determined by trypan blue staining after 30 min treatment with or without CysLT_1_R antagonists, just prior to the initiation of the adhesion assay. (**C**) Representative photographs of crystal violet-stained colonies treated with ZM198,615 (ZM) or Montelukast (Mo) in 6-well plates. (**D**) Relative colony number and (**E**) relative colony size were measured using ImageJ software. The quantitative data shown are the mean ± SEM from three separate experiments. *P<0.05, **P<0.01, ***P<0.001 by paired *t* test or one-way ANOVA.

Cell adhesion is a complex mechanism involved in various of processes of tumor development, such as tumor cell migration, invasion, and angiogenesis [35]. Therefore, we evaluated the effects of CysLT_1_R antagonists on cell adhesion after a short (90 min) exposure of HCT-116 cells. Adherent cells decreased by 28% and 76% when treated with ZM198,615 (50 µM) and Montelukast (25 µM), respectively, compared to DMSO-treated cells (Figure 7A). We did not detect any effect on cell viability, as measured by trypan blue staining (Figure 7B). These findings suggest that CysLT_1_R might be involved in cell adhesion, an important mechanism in the metastatic process of cancer.

The anchorage-independent assay is an established method for testing the tumorigenic ability of cancer cells *in vitro*. A soft agar assay was carried out for HCT-116 cells treated with CysLT_1_R antagonists for 2 weeks (Figure 7C). Figure 7D show a statistically significant reduction of 50 µM ZM198,615-treated colonies (77.9±7.5%) compared to DMSO-treatment. Montelukast treatment showed an even stronger inhibitory effect on colony formation at the lower dosage of 12.5 µM, a reduction of 81.5±12.2% compared to DMSO-treated cells (Figure 7D). We also observed a dose-dependent reduction in colony size for both CysLT_1_R antagonist treatments (Figure 7E). These results suggest the importance of CysLT_1_R in tumor initiation.

### Montelukast Decrease HT-29 and SW-480 Xenograft Tumor Growth

**Figure 8:**
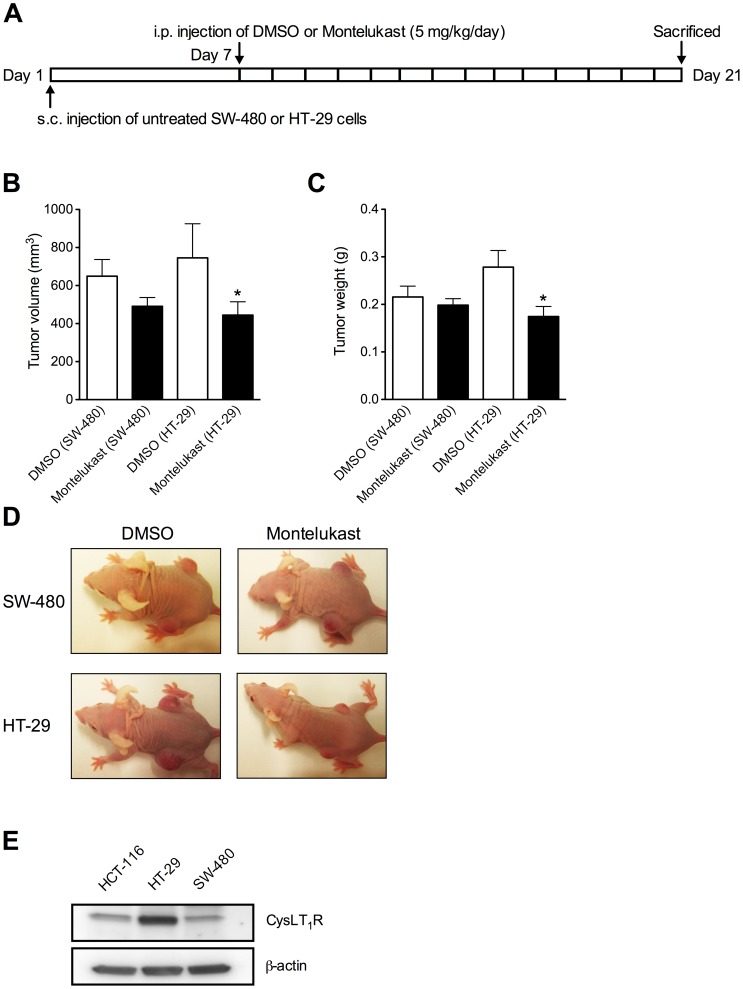
Effects of the CysLT_1_R antagonist Montelukast on HT-29 and SW-480 xenograft tumor growth. (**A**) Experimental protocol; untreated SW-480 or HT-29 cells were subcutaneously injected into both flanks of BalbC (nu/nu) mice. These mice received daily intraperitoneal injections with DMSO or Montelukast (5 mg/kg) for 14 days, starting 7 days after tumor cell inoculation. (**B**) Tumor weight and (**C**) volume at the experimental endpoint (day 21). (**D, E**) Tumor diameters over a 21-day period. (**F**) Representative *in situ* tumor images. The quantitative data shown are the mean ± SEM. *P<0.05. Tumor volume and weight analysis was performed by Student’s *t* test and tumor diameter analysis was performed by two-way ANOVA.

To further evaluate the effects of CysLT_1_R antagonists on cancer growth *in vivo*, we employed two additional human colon adenocarcinoma cell lines, namely HT-29 and SW-480. Seven days after inoculation all mice had tumors in both flanks and the treatments were then initiated. The mice received daily i.p. injections with either DMSO or Montelukast (5 mg/kg) for two weeks (Figure 8A). At the experimental endpoint, day 21, a significant decrease in tumor volume (*P*<0.05; Figure 8B) and tumor weight (*P*<0.05; Figure 7C) were observed for HT-29 xenograft tumors treated with Montelukast (5 mg/day) as compared to DMSO. Similar tendencies were observed for SW-480 tumor xenografts (Figure 8B and C). The less significant response in SW-480 cells could possibly be due to their lower expression of CysLT_1_Rs (Figure 8E). Representative *in situ* tumor images from each treatment group for both colon adenocarcinoma xenograft models can be observed in Figure 8D.

Furthermore, the expression of CysLT_1_R in all three human colon adenocarcinoma cell lines was verified (Figure 8E) and an endogenous production and release of CysLT_1_R substrates, i.e. cysteinyl leukotriens, could also be demonstrated (Table 1).

**Table 1:** Basal expression level of CysLTs in cell culture media of indicated colon cancer cell lines.

Colon cancer cell lines	CysLTs (pg/ml)
HCT-116	204.5±16.17
HT-29	234.9±13.14
SW-480	221.0±33.78

The data shown are the mean ± SEM.

## Discussion

CysLT_1_R has been shown to be upregulated in several types of human cancers, including transitional cell carcinoma (TCC) in the bladder, neuroblastoma, and brain, prostate, breast, and colon cancers [16], [34], [36], [37], [38], [39]. Its increased expression in tumors is also correlated with poor survival in patients with breast or colon cancer [16], [39]. Our previous studies have shown that CysLT_1_R is highly expressed in established colon cancer lines and in colon cancer patients [16], [28].

In the present study, we investigated the functional importance of CysLT_1_R in colon cancer initiation and progression *in vivo* using the HCT-116 xenograft mouse model and two different drug administration regimens.

CysLT_1_R is of functional importance in colon cancer development as demonstrated by the reduced volume and weight of HCT-116 tumors in BalbC (nu/nu) mice challenged with CysLT_1_R antagonists (5 mg/kg/day) after tumor appearance compared to DMSO-treated mice. A moderately decreased expression of the proliferation marker Ki-67 was detected in tumor xenografts of mice challenged with CysLT_1_R antagonists after tumor appearance compared to tumor xenografts of mice challenged with DMSO. At the experimental endpoint, we were unable to detect any statistically significant changes in angiogenesis as determined by immunostaining of CD31.

Animals receiving HCT-116 colon cancer cells pretreated with ZM198,615 before transplantation exhibited tumors of markedly reduced volume and weight. This finding could implicate an important role of CysLT_1_R in the initiation stage of colon cancer. The fact that mice receiving HCT-116 colon cancer cells pretreated with Montelukast did not exhibit any tumors could be due to the potency of this drug. Reported drug potencies, which are the half-maximal inhibitory concentrations for ZM198.615 and Montelukast in terms of inhibiting the binding of [^3^H]-LTD_4_ to guinea lung membranes, are 2.66 nM and 0.64 nM, respectively [40], [41]. The ZM198,615 pre-treated group exhibited tumors with moderately decreased proliferative ability compared to DMSO-treated animals. In contrast to animals receiving ZM198,615 treatment first after tumor appearance, animals from the ZM198,615 pretreated group had significantly smaller vessel formation in their tumors compared to DMSO-treated animals, as determined by CD31 immunostaining. In addition to a significant decrease in vascular size in xenograft tumors of animals transplanted with ZM198,615 pretreated cells, a significant decrease in the expression levels of VEGF was also detected by immunoblotting. A significant decrease in VEGF expression was also observed in tumor samples from the Montelukast-treated group compared to DMSO II tumor samples. Thus, we postulate that CysLT_1_R antagonists impair angiogenesis in colon cancer xenografts, inhibiting their growth.

The effects of CysLTs on vascular responses, which are related to vascular permeability and subsequent plasma extravasation, are mediated via CysLT_1_R [42]. CysLT_1_R antagonists Pranlukast and Montelukast have been shown to reduce vascular permeability by regulating VEGF expression in the lungs of mice with allergen-induced asthma [43]. These antagonists have also been shown to inhibit tumor metastasis in a Lewis lung carcinoma metastasis model by inhibiting the permeability of peripheral capillaries [44]. Interestingly, Montelukast has also been shown to reduce LTD_4_-induced migration of the endothelial cell line EA.hy926 mediated by CysLT_1_Rs via the Erk1/2 pathway [45]. Both proliferation and migration of endothelial cells are needed to form new vessels.

We also demonstrated that CysLT_1_R antagonists ZM198,615 and Montelukast inhibit xenograft tumor growth partly by inducing apoptosis and cell cycle arrest. Increased expression levels of cleaved caspase 3, capase-cleaved product of cytokeratin 18 and p21^WAF/Cip1^ were found both in xenografted tumors of mice transplanted with ZM198,615 pretreated HCT-116 cells, receiving continued treatment from the day of implantation, and in xenografted tumors of mice transplanted with untreated HCT-116 cells, receiving treatment after tumor appearance.

These data were further strengthened *in vitro* by the findings of induced apoptosis and cell cycle arrest at G1 phase in the colon cancer cell line HCT-116 after CysLT_1_R antagonist treatment, as analyzed by flow cytometry. Interestingly, CysLT_1_R antagonist treatment has been shown to inhibit growth of a series of human urological cancer cell lines (e.g., renal cell carcinoma, bladder cancer, prostate cancer, and testicular cancer) by inducing apoptosis [46]. It has also been shown that administration of Montelukast (100 µM) induces early apoptosis in T24 cells, a human TCC cell line, and in three different prostate cancer cell lines [34], [36]. Montelukast has also been shown to induce the intrinsic apoptotic pathway, resulting in cleavage of caspases 3 and 9, and cell cycle arrest in neuroblastoma cell lines [37].

From previous *in vitro* data, it was believed that colon cancer cells were resistant to CysLT_1_R antagonist-induced apoptosis [47]. In the present study compared to previous *in vitro* studies, concerning the colon cancer cell line HCT-116 and detection of apoptosis (i.e., by immunoblotting cleaved caspase 3), Montelukast was used instead of its precursor MK571. Previous results may have reflected differences in drug potency, rather than a connection between nuclear localization of CysLT_1_R and CysLT survival signaling in colon cancer cells. Indeed, Montelukast has been shown to be more potent than MK571 in inhibiting 50% of cell viability (EC_50_) in a broad range of neuroblastoma cell lines [37].

In the present study, we show that the CysLT_1_R antagonists ZM198,615 and Montelukast reduce cell proliferation of the colon cancer cell line HCT-116 in a dose-dependent manner. We have previously shown that ZM198,615 (50 µM) reduces proliferation in a subset of colon cancer cell lines, namely, Caco-2 and SW480 [47]. Another study reported the additive effects of the COX-2 selective inhibitor Celecoxib in reducing the proliferative ability of the colon cancer cell lines HT29 and Caco-2 when combined with either the 5-LOX inhibitor MK886 or the CysLT_1_R antagonist LY171883. They also reported that the combined drug treatment induced apoptosis in HT29 and Caco-2 cells, whereas neither of these compounds alone had any effect [48].

Furthermore, using two additional human adenocarcinoma cell lines, namely SW-480 and HT-29, we were able to strengthen the *in vivo* data and demonstrate the ability of the CysLT_1_R antagonist Montelukast to inhibit colon cancer xenograft growth. All three colon adenocarcinoma cell lines used in the *in vivo* xenograft studies were shown capable of endogenous production and release of cysteinyl leukotrienes in culture media. We have demonstrated that CysLT_1_R antagonists alone inhibit cell proliferation and induce cell apoptosis *in vitro*. Therefore, it is likely that the cysteinyl leukotriene levels produced *in vitro* by the HCT-116 cell line are sufficient to induce cell growth and survival. However, if the production and release of cysteinyl leukotrienes is sufficient to act in an autocrine manner and convey a self-growth of xenograft tumors is yet to be demonstrated. Even though non-leukocytes cells do not express high levels of 5-lipooxygenase and FLAP and are therefore not believed to have appreciable endogenous production of leukotrienes, there is a possibility of transcelllular biosynthesis [49]. This mechanism and the presence of host leukocytes in the vicinity of the xenograft tumors could also be a potential secondary source of cysteinyl leukotrienes.

The COX pathway is the most extensively studied of the eicosanoid pathways in terms of chemoprevention and/or treatment of colon cancer. However, the cardiovascular side effects associated with prolonged treatment with NSAIDs and selective COX-2 inhibitors have raised some great concerns, and other approaches such as inhibition of 5-LOX activity are currently being explored extensively. Notably, the antitumor effects of the selective COX-2 inhibitor Celecoxib in colon cancer cells are augmented when combined with inhibition of 5-LOX activity with the FLAP inhibitor MK886 [48]. The dual inhibition of COX-2 and 5-LOX has also been shown to suppress cigarette smoke-promoted growth of colon cancer in a nude mouse xenograft model [50]. In these studies, a shunt toward either of the pathways was detected when one pathway was targeted, except for the latter experiment in which a shunt was only observed when inhibiting COX-2 with Celecoxib.

The colon cancer cell line HCT-116 does not constitutively express COX-2; approximately one-half of the 84 colorectal adenocarcinoma specimens examined in a tissue array study were estimated to have elevated expression of COX-2 [16]. The HCT-116 nude mice xenograft model employed in the present study, however, can represent a targeted therapy investigation. Colorectal cancer patients lacking increased expression levels of COX-2 would not be expected to display a possible “shunt” of arachidonic acid metabolism into the COX-2 pathway and would be expected to respond well to CysLT_1_R antagonist treatment.

To our knowledge, this is the first report of the anti-growth abilities of CysLT_1_R antagonists on colon cancer tumors *in vivo* in a xenograft model. At present the majority of oncology drug development *in vivo* relies on transplantable human tumor xenograft models to predict clinical activity of novel compounds. The orthotopic xenograft model has in may situations distinct advantages such as providing a more appropriate tumor environment and enabling studies of tumor metastasis. A subcutaneous xenograft model on the other hand provides a rapid, easy reproducible and less laborious method [51]. The reason why we have chosen the latter model for our present study was that is best suited for investigation of how treatment with CysLT_1_R antagonists affects the kinetics of colon cancer growth *in vivo*.

In summary, our results suggest that targeting CysLT_1_R can prevent colon cancer initiation and/or progression, as demonstrated in a xenograft mouse model, primarily via reduction of tumor cell proliferation and induction of apoptosis. Our previous studies indicated that the inflammatory receptor CysLT_1_ has a prognostic value, and the present *in vivo* data highlight the prospect of this receptor as a target in colon cancer therapy. In particular, patients with tumors expressing low levels of COX-2 and high levels of CysLT_1_R could benefit from targeted treatment with a CysLT_1_R antagonist. Such an antagonists could also be used in a combination treatment strategy with COX inhibitors in patients with tumors expressing high COX-2 level to provide better treatment efficacy. However, these postulations require further study.
